# Region-Specific Vulnerability of the Amygdala to Injury-Induced Spreading Depolarization

**DOI:** 10.3390/biomedicines10092183

**Published:** 2022-09-03

**Authors:** Mariia P. Smirnova, Tatiana M. Medvedeva, Irina V. Pavlova, Lyudmila V. Vinogradova

**Affiliations:** Institute of Higher Nervous Activity and Neurophysiology, Russian Academy of Sciences, 117485 Moscow, Russia

**Keywords:** spreading depolarization, intracerebral micro-injury, basolateral amygdala, centromedial amygdala

## Abstract

Spreading depolarization (SD), a self-propagated wave of transient depolarization, regularly occurs in the cortex after acute brain insults and is now referred as an important diagnostic and therapeutic target in patients with acute brain injury. Here, we show that the amygdala, the limbic structure responsible for post-injury neuropsychological symptoms, exhibits strong regional heterogeneity in vulnerability to SD with high susceptibility of its basolateral (BLA) region and resilience of its centromedial (CMA) region to triggering SD by acute focal damage. The BLA micro-injury elicited SD twice as often compared with identical injury of the CMA region (71% vs. 33%). Spatiotemporal features of SDs triggered in the amygdala indicated diverse patterns of the SD propagation to the cortex. Our results suggest that even relatively small cerebral structures can exhibit regional gradients in their susceptibility to SD and the heterogeneity may contribute to the generation of complex SD patterns in the injured brain.

## 1. Introduction

Spreading depolarization (SD) is a self-propagated wave of transient mass depolarization associated with the breakdown of ion homeostasis, excitotoxic edema, complex cerebrovascular changes, etc. [1]. Spontaneous SDs regularly occur after acute brain insults (ischemic stroke, intracerebral hemorrhage, traumatic brain injury). It is believed that injury-induced SDs promote the focal lesion expansion and development of secondary brain damage. Nowadays, SD is referred to as an important prognostic and therapeutic target in patients with acute brain injury. To date, the majority of SD studies have focused on the cortex, but it is known that SD can be triggered in almost all brain structures, although with different ability [1,2,3]. For example, forebrain structures easily generate SD in response to elevated potassium concentration, electroshock or anoxia, whereas the hypothalamus and the brainstem sites are resistant to SD generation under the same conditions [2,4]. The forebrain/brainstem discrepancy in sensitivity to SD is supposed to correspond to vulnerability of the regions to traumatic brain injury [4]. Regional differences in susceptibility to SD were found in the cortex of animals and humans that are thought to underlie the triggering of migraine aura in specific cortical areas [5]. Mechanisms of the region-specific vulnerability to SD remain obscure. It has been suggested that these mechanisms may include regional variability of the Na+/K+ pump strength, neuronal/glial density, myelination, the state of glial transport, neurotransmitter systems, etc. [1,3,5]. 

Recently we developed a method of SD induction via standard local micro-injury of the brain parenchyma [6]. Using the method, we found that a rather small volume of tissue damage (0.3 mm^3^) was sufficient to trigger SD in the cortex and amygdala of unanesthetized rats [6,7]. However, the amygdala damage triggered SD with significantly lower probability than the identical damage of the cortex did [6]. Reasons for the reduced vulnerability of the amygdala to SD remained unclear. 

The amygdala is a highly nonhomogeneous (both anatomically and neurochemically) cluster of nuclei involved in pain processing and emotional behavior [8]. The limbic structure is thought to be responsible for post-injury neurobehavioral symptoms. The amygdala may be injured in strokes or other brain damage that affects the temporal lobe. We hypothesized that the functional heterogeneity of the amygdala may affect its susceptibility to injury-induced SD. In the present study we examined intra-amygdala vulnerability to SD and found a striking difference in the sensitivity to injury-induced SD between the centromedial and the basolateral regions of the amygdala.

## 2. Materials and Methods

### 2.1. Subjects

Thirty-eight male Wistar rats (350–450 g, Scientific center for Biomedical Technologies of the Federal Medical and Biological Agency, Russia) were housed in a temperature-controlled vivarium (22 °C ± 2 °C, a 12-h light/dark cycle, lights on at 08.00 h) with food and water ad libitum. Animals were kept 5 per cage before surgery and individually after surgery. All experimental procedures were conducted in accordance with the ARRIVE guidelines and the Directive 2010/63/EU for animal experiments. The study protocol was approved by the Ethics Committee of the Institute of Higher Nervous Activity and Neurophysiology of the Russian Academy of Sciences. Every effort was made to minimize animal suffering and to ensure reliability of the results.

### 2.2. Stereotaxic Surgery

Under chloral hydrate anesthesia (400 mg/kg, i.p. AppliChem, Germany), rats were implanted with bilateral/unilateral stainless steel guide cannulas (23 gauge) aimed at the amygdala (AP: −2.76, ML: −4.8 mm DV: −7.5 mm) [9]. Recording electrodes (silver or nichrome, diameter of 0.25–0.30 mm) were implanted in the frontal (AP: +1.2, ML: ±2.3 mm, DV: −1.8) and occipital (AP: −5.88, ML: ±3.5 mm, DV: −1.5 mm) cortices. The tips of the intracortical recording electrodes were located at a depth of 0.5–1.2 mm below the cortical surface. The guide cannulas and electrodes were fixed on the skull with acrylic dental plastic. A 30-gauge stylus of the same length as the guide cannula was inserted into it to prevent clogging. Experiments started two weeks post-surgery. All animals were pre-handled and habituated to the stylus removal daily at three–four days before the start of the experiments.

### 2.3. Micro-Injury of the Amygdala and SD Recording

Awake, freely moving rats were individually placed in a shielded experimental chamber (60 × 40 × 40 cm) and the implanted connector was attached to the recording cable. After a 5-min habituation period and a 10-min baseline (pre-injury) recording of wideband cortical activity (0–100 Hz, 1 kHz sampling rate), a bilateral/unilateral amygdala micro-injury was induced. SD always occurred at the ipsilesional side. During the injury induction, the awake rat was gently handled and the injection cannula, extending 1.0 mm from the tip of the guide cannula, was inserted. Cortical activity was recorded for 15 min post-injury using a four-channel, high-input impedance (1 gΩ) dc amplifier and a/d converter (E14-440, L-Card, Russia) with simultaneous video monitoring of behavior. SD occurrence was identified based on detection of characteristic, abruptly developing, high-amplitude (larger 2 mV) negative shift of the slow potential. SD latency was determined as the time delay between the amygdala injury and SD appearance at the recording cortical sites.

### 2.4. Histology

For histological verification of amygdalar injury and localization of recording electrodes, animals were overdosed with chloral hydrate and perfused intracardially with 0.9% saline after the end of the experiments. Their brains were removed, stored in 10% formalin for 48 h, sectioned in coronal 50-μm slices and stained with 0.1% cresyl violet. The slices were used to verify the cannula and recording electrode positions and to evaluate the volume of tissue damage produced by the needle insertion. Localization and volume of the damage were reconstructed on stereotaxic atlas templates from Paxinos and Watson [9] using a light microscope. We also used the 2D Rat Brain Atlas (http://labs.gaidi.ca/rat-brain-atlas/ (accessed on 19 February 2017)) to re-examine our data regarding injury location and electrode placement.

### 2.5. Statistical Analysis

Statistical analyses were performed using Statistica software 8.0 (StatSoft). Spearman’s correlation was used to assess the correlation between probability of SD and anterior–posterior, medial–lateral, dorsal–ventral positions of the micro-injury. Regional differences in SD occurrence and lesion size were estimated using the Fisher’s exact test and Mann–Whitney U test, respectively. The significance was set at *p* < 0.05. Python language was used for color-coded visualization of the SD occurrence for different locations of intra-amygdala injuries.

## 3. Results 

**Figure 1:**
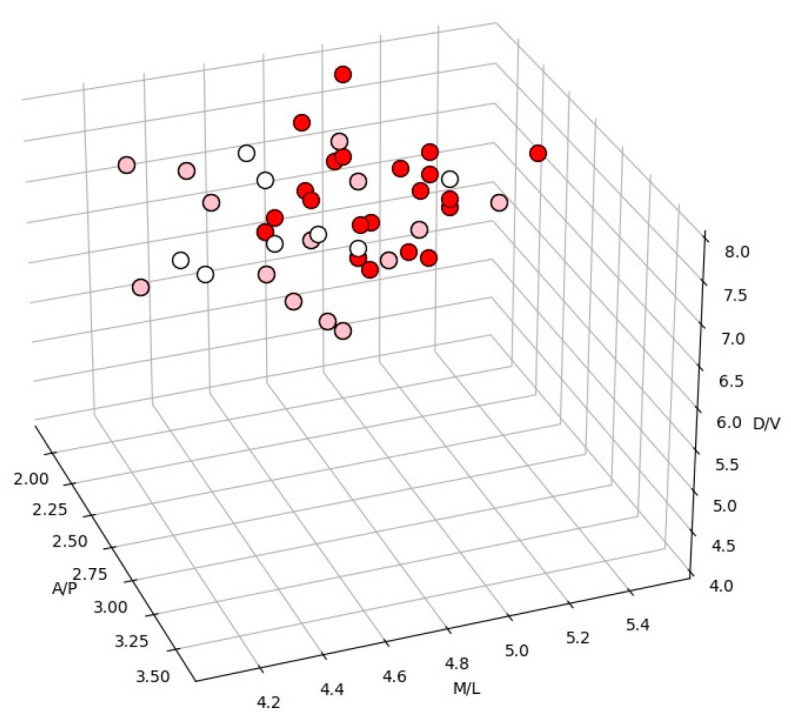
Color-coded visualization of SD occurrence for different locations of intra-amygdala injuries. The red, pink and white dots mark 100% (2/2), 50% (1/2) and 0% (0/2) occurrence of injury-induced SD in two repeated tests.

**Figure 2:**
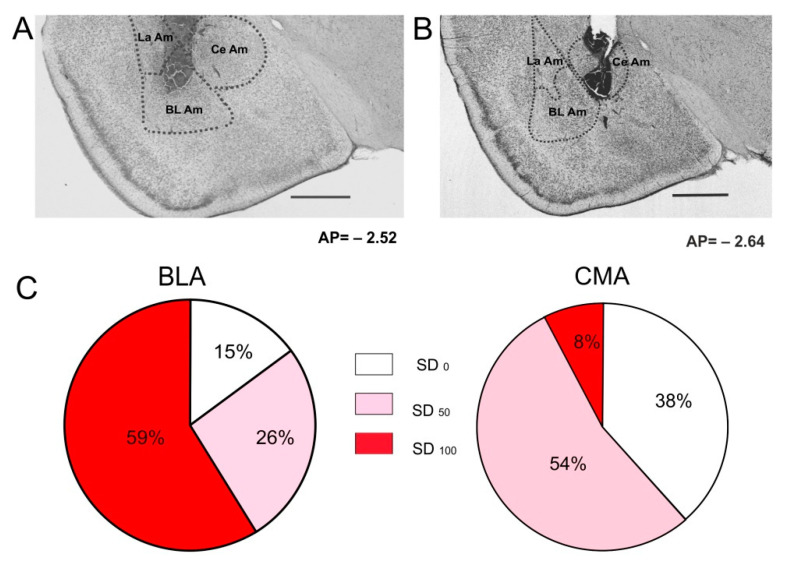
Photomicrographs of the typical amygdala lesion produced by micro-injury of the basolateral (**A**) and centromedial (**B**) regions of the amygdala. Scale bar is 1 mm. BLA—basolateral amygdala, Ce Am—central amygdala, La Am—lateral amygdala. (**C**) Pie charts showing probability of SD-triggering by two injuries (100%, 50% and 0%) of BLA and centromedial (CMA) amygdala regions.

SD was never recorded before the injury of the amygdala; however, a single SD episode was recorded after most of the injuries. A total of 47 out of 55 injured sites were localized within the amygdala and included in the analysis. Figure 1 shows the color-coded visualization of SD occurrence for different locations of the intra-amygdala injuries. As can be seen, more lateral sites of the amygdala showed more reliable triggering of SD by the injury. Spearman’s correlation analysis showed a positive correlation between SD probability and the medial–lateral position of the micro-injury (r = 0.54, *p* < 0.05); however, no correlation was seen between SD occurrence and anterior–posterior (r = −0.16, *p* > 0.05) or dorsal–ventral positions (r = −0.14, *p* > 0.05). Based on the results, all of the injured sites were attributed to two groups, according to their localization within the centromedial (CMA) or basolateral (BLA) regions of the amygdala (Figure 2A,B). There was no significant difference in lesion volumes between the BLA and CMA groups (0.27 ± 0.04 mm^3^ for BLA and 0.35 ± 0.04 mm^3^ for CMA, *p* > 0.05). The occurrence of SD after the injury of the BLA and CMA regions is shown in Figure 2C. The BLA injury reliably induced SD in two repeated tests, whereas CMA injuries usually elicited SD in neither or in only one of the two tests (Figure 2C). In total, 71% (50/70) of BLA injuries and 33% (8/24) of CMA damage triggered SD (*p* = 0.0014, two-tailed Fisher’s test). 

**Figure 3:**
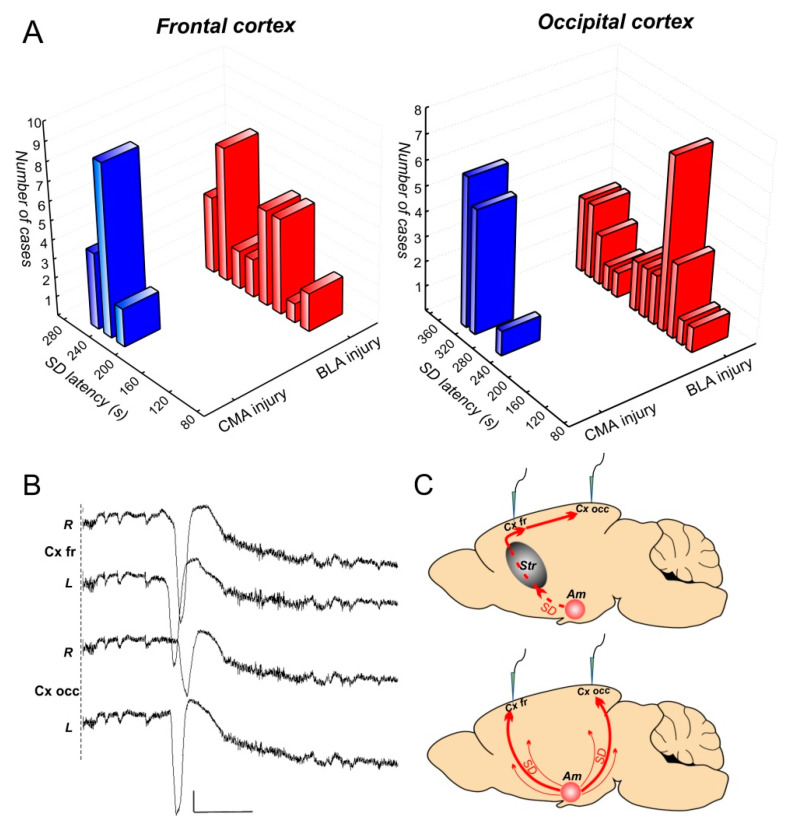
(**A**) Latencies of SD appearance in the frontal (Cx fr) and occipital (Cx occ) cortices after CMA and BLA injury; (**B**) DC recording of SDs induced by the bilateral injury of the BLA in the frontal and occipital cortical areas of the two hemispheres; scale bars—100 s and 2 mV; negativity is directed downward; (**C**) schemes of the two pathways of SD propagation from the amygdala to the cortex via the striatum and frontal pole (above) or via the temporal cortex (bottom). Str—the striatum, Am—the amygdala.

SDs triggered by the injury of both the amygdala regions propagated to the cortex, and a long latency (3–6 min) of the SD propagation suggested its non-synaptic mechanisms (Figure 3A). After CMA injury, SD appeared initially in the frontal cortex at 230 s (median, range 220–260 s) post-injury, and approximately 100 s later (at 330 s, range 243–340 s) in the occipital cortex. This pattern of SD spread suggested that SD ignited in the CMA, propagated to the cortex via the rostral pole and then moved over the dorsal cortex in the rostro-caudal direction. Given the distance between frontal and occipital recording electrodes of 8 mm, the velocity of SD propagation over the cortex was about 5 mm/min, which is similar to the data from our previous study in awake rats [6]. After BLA injury, SD appeared in the cortex, either with a long latency, as SDs induced in the CMA, or with a shorter delay, and almost simultaneously to the frontal (210 s, range 105–260 s) and occipital (220 s, range 105–360 s) cortices (Figure 3A). A DC recording of SDs induced by bilateral injury of the BLA is shown in Figure 3B. The pattern suggested frequent propagation of SD from BLA to the cortex directly via the temporal cortex (Figure 3C). 

The injury did not induce any immediate behavioural response per se but propagation of the injury-induced SD within the cortico-striatal system was accompanied by mild behavioural changes described in our previous study [6]. 

## 4. Discussion

Our study shows a high susceptibility of the basolateral (BLA) region of the brain and resilience of the centromedial (CMA) region to triggering SD by acute focal damage. The striking difference in vulnerability to injury-induced SD between the CMA and BLA regions of the amygdala is in line with the data regarding the abilities of these regions to sustain SD propagation from the cortex, with development of high-amplitude SDs in BLA and no SD in the CMA region [10,11]. Mechanisms of SD initiation are not completely clear yet [1]. It is generally thought that massive neuronal depolarization and an increase in extracellular potassium concentration above a critical value are key events for the initiation of SD [2,3]. The present study shows that the CMA and BLA damage of identical sizes triggered SD with different ability. Therefore, apart from membrane depolarization and an increased extracellular potassium concentration, additional factors seem to be required for SD triggering in the amygdala, and we believe that one of these factors is the regenerative glutamate release. No significant differences in neuronal density and neuroglial ratio have been described between the amygdala regions, as in [12], but the regions significantly differ in patterns of glutamatergic and GABAergic transmission. The BLA is known to possess cortical-like features and more than 80% of BLA neurons are excitatory glutamatergic neurons [13]. CMA exhibits striatal-like features and contains a strong inhibitory network with multiple GABAergic local and projection neurons [14]. Dense GABA neurons were found in the central nucleus, whereas only scattered GABA neurons were found in the rest of the amygdala [15]. The difference in the glutamate/GABA ratio between the CMA and BLA regions may underlie the difference in vulnerability of the regions to injury-induced SD found in our study. The idea also agrees with the data regarding the critical role of AMPA and NMDA receptors for triggering SD (CBF changes and neuronal firing) by mechanical stimulation of the cortex [16]. Excessive glutamatergic activation is known as an important prerequisite for SD ignition in migraine [17]. Glutamatergic mechanisms and local excitatory networks may be especially important for the initiation of the positive feedback cycle underlying SD in intact neuronal tissue remote from injured regions. However, it is known that other neurotransmitter systems are also differentially expressed in the CMA and BLA regions [18,19]; therefore, a potential role of these systems cannot be excluded and this deserves further investigation.

SD is known to propagate over the grey matter, irrespective of anatomic, functional or synaptic architecture, and both gyrencephalic and lissencephalic cortices support complex SD propagation patterns. It has been previously shown that SD induced in the cortico-striatal system of rodents can non-synaptically propagate without detriment to all structures of the system. Cortical SD spreads to the amygdala and striatum [11,20,21]; striatal SD propagates to the amygdala and cortex [2,10,20]; SD induced by the amygdala injury always invades the cortex and striatum [6,7]. Striatal SD can propagate to the cortex via two different pathways—via rostral (more reliable) and temporal transit points due to minimal anatomical barriers at the interface regions [20]. Now we have shown that SD triggered in the amygdala propagates to the cortex via similar ways—(1) the striatum and rostral pole and/or (2) the temporal cortex (Figure 3C), and we also observed an apparent preference for spreading through the rostral pole. SD triggered in the BLA region used both ways while SD induced in the CMA region propagated only through the striatum and never spread via the temporal cortex. Structurally and functionally, the CMA and BLA are referred to as the ventral extension of the striatum and the nuclear extension of the cortex, respectively [15,19]. The close relationship of BLA with the cortex is likely to determine the possibility of the direct spread of SD triggered in the BLA to the cortex.

SD is a reliable post-injury symptom and the amygdala plays a key role in cognitive/emotional processing; therefore, the high vulnerability of some amygdala regions to injury-induced SD could underlie cognitive/emotional symptomatology in patients with acute brain injury. The findings of the present study may be important for understanding variable clinical consequences of brain damage that did not primarily affect the cerebral cortex. Given the well-known long-lasting effects of SD on neuronal excitability, metabolism and cerebral perfusion [2,3], the propagation of SD induced by a focal injury to remote brain sites may contribute to a distant clinical deficit (acute traumatic diaschisis). 

## Data Availability

Data is contained within the article.

## Abbreviations

SD	Spreading depolarization
BLA	Basolateral amygdala
CMA	Centromedial amygdala
AP	Anterior–posterior
ML	Medial–lateral
DV	Dorsal–ventral

## References

[1] Andrew R.D., Hartings J.A., Ayata C., Brennan K.C., Dawson-Scully K.D., Farkas E., Herreras O., Kirov S.A., Müller M., Ollen-Bittle N. (2022). The Critical Role of Spreading Depolarizations in Early Brain Injury: Consensus and Contention. Neurocrit. Care.

[2] Bures J.O., Buresova J.K. (1974). The Mechanism and Applications of Leao’s Spreading Depression of EEG Activity.

[3] Somjen G.G. (2001). Mechanisms of Spreading Depression and Hypoxic Spreading Depression-Like Depolarization. Physiol. Rev..

[4] Andrew R.D., Hsieh Y.-T., Brisson C.D. (2016). Spreading depolarization triggered by elevated potassium is weak or absent in the rodent lower brain. J. Cereb. Blood Flow Metab..

[5] Bogdanov V.B., Middleton N.A., Theriot J.J., Parker P., Abdullah O.M., Ju Y.S., Hartings J.A., Brennan K.C. (2016). Susceptibility of Primary Sensory Cortex to Spreading Depolarizations. J. Neurosci..

[6] Vinogradova L.V., Rysakova M.P., Pavlova I.V. (2020). Small damage of brain parenchyma reliably triggers spreading depolarization. Neurol. Res..

[7] Rysakova M.P., Pavlova I.V., Vinogradova L.V. (2022). Spreading depolarization induced by amygdala micro-injury prevents disruption of fear memory extinction in rats. Behav. Brain Res..

[8] Gafford G., Ressler K. (2016). Mouse models of fear-related disorders: Cell-type-specific manipulations in amygdala. Neuroscience.

[9] Paxinos G., Watson C. (1982). The Rat Brain in Stereotaxic Coordinates.

[10] Fifková E. (1966). Spreading depression in subcortical structures in rabbit. Brain Res..

[11] Fifková E., Syka J. (1964). Relationships between cortical and striatal spreading depression in rat. Exp. Neurol..

[12] García-Amado M., Prensa L. (2012). Stereological Analysis of Neuron, Glial and Endothelial Cell Numbers in the Human Amygdaloid Complex. PLoS ONE.

[13] McDonald A.J., Aggleton J. (1992). Cell types and intrinsic connections of the amygdala. The Amygdala: Neurobiological Aspects of Emotion, Memory, and Mental Dysfunction.

[14] Butler R.K., Ehling S., Barbar M., Thomas J., Hughes M.A., Smith C.E., Pogorelov V.M., Aryal D.K., Wetsel W.C., Lascelles B.D.X. (2017). Distinct neuronal populations in the basolateral and central amygdala are activated with acute pain, conditioned fear, and fear-conditioned analgesia. Neurosci. Lett..

[15] Swanson L.W., Petrovich G.D. (1998). What is the amygdala?. Trends Neurosci..

[16] Holland P., Akerman S., Goadsby P.J. (2010). Cortical spreading depression-associated cerebral blood flow changes induced by mechanical stimulation are modulated by AMPA and GABA receptors. Cephalalgia.

[17] Capuani C., Melone M., Tottene A., Bragina L., Crivellaro G., Santello M., Casari G., Conti F., Pietrobon D. (2016). Defective glutamate and K^+^ clearance by cortical astrocytes in familial hemiplegic migraine type 2. EMBO Mol. Med..

[18] Ben-Ari Y., Zigmond R., Shute C., Lewis P. (1977). Regional distribution of choline acetyltransferase and acetylcholinesterase within the amygdaloid complex and stria terminalis system. Brain Res..

[19] LeDoux J. (2007). The amygdala. Curr. Biol..

[20] Vinogradova L., Koroleva V., Bures J. (1991). Re-entry waves of Leao’s spreading depression between neocortex and caudate nucleus. Brain Res..

[21] Samotaeva I., Tillmanns N., van Luijtelaar G., Vinogradova L. (2013). Intracortical microinjections may cause spreading depression and suppress absence seizures. Neuroscience.

